# Update on oral-facial-digital syndromes (OFDS)

**DOI:** 10.1186/s13630-016-0034-4

**Published:** 2016-05-02

**Authors:** Brunella Franco, Christel Thauvin-Robinet

**Affiliations:** Telethon Institute of Genetics and Medicine (TIGEM), Via Campi Flegrei 34, Pozzuoli, 80078 Naples, Italy; Medical Genetics, Department of Medical Translational Sciences, University of Naples Federico II, Naples, Italy; EA GAD, IFR Santé–STIC, Université de Bourgogne, Dijon, France; Centre de Référence Maladies Rares « Anomalies du Développement et Syndromes malformatifs » de l’Est, Centre de Génétique et Pédiatrie 1, Hôpital d’Enfants, CHU Dijon, Dijon, France

**Keywords:** Cilia, OFDS, Developmental disorders

## Abstract

Oral-facial-digital syndromes (OFDS) represent a heterogeneous group of rare developmental disorders affecting the mouth, the face and the digits. Additional signs may involve brain, kidneys and other organs thus better defining the different clinical subtypes. With the exception of OFD types I and VIII, which are X-linked, the majority of OFDS is transmitted as an autosomal recessive syndrome. A number of genes have already found to be mutated in OFDS and most of the encoded proteins are predicted or proven to be involved in primary cilia/basal body function. Preliminary data indicate a physical interaction among some of those proteins and future studies will clarify whether all OFDS proteins are part of a network functionally connected to cilia. Mutations in some of the genes can also lead to other types of ciliopathies with partially overlapping phenotypes, such as Joubert syndrome (JS) and Meckel syndrome (MKS), supporting the concept that cilia-related diseases might be a continuous spectrum of the same phenotype with different degrees of severity. To date, seven of the described OFDS still await a molecular definition and two unclassified forms need further clinical and molecular validation. Next-generation sequencing (NGS) approaches are expected to shed light on how many OFDS geneticists should consider while evaluating oral-facial-digital cases. Functional studies will establish whether the non-ciliary functions of the transcripts mutated in OFDS might contribute to any of the phenotypic abnormalities observed in OFDS.

**Electronic supplementary material:**

The online version of this article (doi:10.1186/s13630-016-0034-4) contains supplementary material, which is available to authorized users.

## Background

The oral-facial-digital syndromes (OFDS) represent a group of rare developmental disorders characterized by abnormalities of the face, oral cavity and digits. Additional signs involving the central nervous system (CNS), and visceral organs, such as the kidney, are also frequently observed. The first case presenting this condition was reported in 1941 [1] and since then a number of different OFDS types with overlapping phenotypes have been described [2, 3] (Table 1). Among the different types, OFD type I is the most frequently observed and can be easily recognized by its typical X-linked dominant male-lethal pattern of inheritance in familial cases. Most of the other OFDS are transmitted as autosomal recessive syndromes or represent sporadic cases. In the last few years, 11 genes responsible for OFDS have been identified allowing a better clinical and genetic definition for this heterogeneous condition. This review will focus on the most recent findings on OFDI, III, IV, VI, IX, XIV and two unclassified OFD subtypes. For all other OFDS please refer to [3]. On the basis of the recent molecular data, we can distinguish (1) two more common types (OFDI and OFDVI), for which the causative genes have been identified; (2) four rare subtypes for which the causative gene has also been identified (OFDIII, OFDIV, OFDIX and OFDXIV), thus allowing molecular diagnosis; (3) two unclassified rare OFD subtypes whose causative genes have been identified but that still require further clinical and molecular validation and (4) additional unclassified OFDS which still await molecular characterization and further definition (Table 1). Table 2 reports a clinical summary of the different OFDS clearly identified to date.

**Table 1:** Classified OFDS

OFD subtypes	MIM#	Altern SYMB	Aliases	Gene	REF*/Notes
OFDI	311200	OFDSI; OFD1	Orofaciodigital type I; Oral-facial-digital type I; Papillon-Leage/Psaume syndrome	*OFD1*	[7, 13]
OFDII	252100	OFDSII; OFD2	Orofaciodigital type II; Oral-facial-digital type II; Mohr syndrome	–	[3]
OFDIII	258850	OFDSIII; OFD3	Orofaciodigital type III; Oral-facial-digital type III: Sugarman syndrome	*TMEM231*	[29]
OFD IV	258860	OFDSIV; OFD4	Orofaciodigital type IV; Oral-facial-digital type IV; Mohr-Majewski Baraitser syndrome	*TCTN3*	[32]
OFDV	174300	OFDSV; OFD5	Orofaciodigital type V; Oral-facial-digital type V Thurston syndrome	–	[3] /Indian origin
OFDVI	277170	OFDSVI; OFD6	Orofaciodigital type VI; Oral-facial-digital type VI Varadi syndrome	*TMEM216* *OFD1*, *C5ORF42 TMEM107*	[24, 34–37]
OFDVII	608518	OFDSVII; OFD7	Orofaciodigital type VII; Oral-facial-digital type VII		[3]
OFDVIII	300484	OFDSVIII; OFD8	Orofaciodigital type VIII; Oral-facial-digital type VIII; Edwards syndrome	–	[3]
OFDIX	258865	OFDSIX; OFD9	Orofaciodigital type IX; Oral-facial-digital type IX	*TBC1D32* *SCLT1*	[41]
OFDX		OFDSX; OFD10	Orofaciodigital type X; Oral-facial-digital type X; Figuera syndrome	–	[3]
OFDXI		OFDXI; OFD11	Orofaciodigital type XI; Oral-facial-digital type XI; Gabrielli syndrome	–	[3]
OFDXII		OFDXII; OFD12	Orofaciodigital type XII; Oral-facial-digital type XII; Moran Barroso Syndrome	–	[3]
OFDXIII		OFD XIII; OFD13	Orofaciodigital type XIII; Oral-facial-digital type XIII; Degner syndrome	–	[3]
OFDXIV	615948	OFDXIV; OFD14	Orofaciodigital type XIV; Oral-facial-digital type XIV;	*C2CD3*	[43]
Unclassified OFD				*WDPCP*	[44]
Unclassified OFD				*DDX59*	[47]

* References for disease gene identification and/or review discussing the main features of the disease

**Table 2:** Clinical features observed in OFD syndromes

LOCUS	Inheritance	Oral features	Facial features	Hands anomalies	Feet anomalies	Skin/Hair features	Renal features	Cardiac features	Cerebral features	Skeletal features	Other abnormalities	Main references
OFD I	X-linked dominant (lethal in males)	Gingival frenulae Lingual hamartomas Cleft/lobulated tongue Cleft palate	Hypertelorism Cleft lip Pseudocleft of the upper lip	Brachydactyly Clinodactyly Polydactyly	Preaxial polydactyly	Miliae Alopecia	Polycystic kidney disease	–	Corpus callosum agenesis, cerebellar hypoplasia	–	Intellectual disability (50 %), cystic ovary and liver	[7, 64, 65]
OFD II	Autosomal recessive	Gingival frenulae Lingual hamartomas Cleft/lobulated tongue Cleft palate	–	Brachydactyly Clinodactyly Polydactyly	Broad hallux Pre/postaxial polydactyly	Thick hair	–	Rare	Porencephaly, Hydrocephaly	Median Y-shaped metacarpal	–	[3]
OFD III	Autosomal recessive	Bifid uvula Lingual hamartomas Lobulated tongue Tooth hypoplasia	Hypertelorism Bulbous nose Low-set ears	Postaxial polydactyly	Postaxial polydactyly	–	End stage Renal failure I–II decade of life	–	Cerebellar vermis hypoplasia. DW malformation with cystic dilation of the IV ventricle. Myoclonia/Eye movement	–	Pectus excavatum Severe intellectual disability	[28, 29]
OFD IV	Autosomal recessive	Gingival frenulae Lingual hamartomas Lobulated tongue Cleft palate	Epicanthus Micrognathia Low-set ears	Brachydactyly Clinodactyly Pre/postaxial polydactyly	Pre/postaxial polydactyly	–	Renal cysts	–	Porencephaly, Occipital encephalocele, Agenesis of corpus callosum, Vermis hypoplasia with MTS	Pectus excavatum Tibial abnormalities	Short stature, Variable intellectual disability	[3, 32]
OFD V	Autosomal recessive	Gingival frenulae (rare)	Midline cleft lip	Postaxial polydactyly	Postaxial polydactyly	–	–	–	–	–		[3]
OFD VI	Autosomal recessive	Gingival frenulae Lingual hamartomas Lobulated tongue Cleft palate	Hypertelorism Cleft lip	Brachydactyly Clinodactyly Syndactyly Median/Postaxial polydactyly	Broad hallux Preaxial polydactyly	–	Renal genesis Renal dysplasia	Rare	Vermis hypoplasia with MTS	Median Y-shaped metacarpal	Variable intellectual disability	[34, 36, 66]; [37]
OFD VII	X-linked dominant	Gingival frenulae Lingual hamartomas Cleft palate	Hypertelorism Cleft lip Asymmetry	Clinodactyly	–	–	Polycystic kidney disease	–	–	–	Moderate intellectual disability	[3]
OFD VIII	X-linked recessive	Gingival frenulae Lingual hamartomas Lobulated tongue Epiglottis hypoplasia	Midline cleft lip Telecanthus Large nose	Bifid thumb Postaxial polydactyly	Preaxial polydactyly	–	–	–	–	Tibia and radius hypoplasia	Psychomotor delay Precocious lethality	[67]
OFD IX	Autosomal recessive	Gingival frenulae Lingual hamartomas Lobulated tongue, Cleft palate	Midline cleft lip Synophrys	Brachydactyly Clinodactyly Polydactyly	Bifid toes	–	–	SD	–	–	Short stature, Microphthalmia, Coloboma	[3, 68]
OFD X	Sporadic	Gingival frenulae Cleft palate	Telecanthus Flat nasal root Retrognathia	Oligodactyly Preaxial polydactyly	–	–	–	–	–	Short 4 limbs Bilateral short radius, Fibular agenesis	–	[69]
OFD XI	Sporadic	Gingival frenulae Cleft palate	Hypertelorism Auricular pits Blepharophimosis	Postaxial polydactyly	Postaxial polydactyly	–	–	–	Ventricular dilatation	Odontoid hypoplasia, Vertebral abnormalities	Deafness, severe intellectual disability, behavioural troubles	[70]
OFD XII	Sporadic	Gingival frenulae Bifid tongue Supernumerary teeth	Macrocephaly Hypertelorism	Pre/postaxial polydactyly	Preaxial polydactyly Club feet	–	–	Septum hypertrophy	Sylvius aqueduct stenosis, corpus callosum agenesis, vermis hypoplasia, myelomeningocele	Short tibiae, Central Y-shaped metacarpal	–	[71]
OFD XIII	Sporadic	Lingual hamartomas	Cleft lip	Brachydactyly Clinodactyly Syndactyly	Brachydactyly Clinodactyly Syndactyly	–	–	Mitral and tricuspid valve dysplasia	Leucoaraïosis	–	Neuropsychiatric troubles, Epilepsy	[72]
OFD XIV	Autosomal recessive	Gingival frenulae, Lingual hamartomas Cleft/lobulated tongue, Cleft palate	Telecanthus	Postaxial polydactyly	Duplication of hallux	–	–	–	Corpus callosum agenesis Vermis hypoplasia with MTS	–	Severe microcephaly Micropenis	[43]
Unclassified OFD	Autosomal recessive	Lobulated tongue Cleft palate	Median cleft lip	Postaxial polydactyly	NA	Thick hair	Fused kidneys	TOF VSD	Corpus callosum agenesis		Moderate intellectual disability. Hirschsprung disease	[47]
Unclassified OFD	Autosomal recessive	Lingual hamartomas	–	Postaxial polydactyly	Duplication of hallux	–	–	Coarctation of the aorta	–	5^th^ Y-shaped metacarpal	–	[44]

## Review

### OFD type I syndrome (OFDI)

OFDI was described in 1954 [4] and further defined in 1962 [5]. It has an estimated incidence of 1:50,000 live births [6] and it has been reported in different ethnic backgrounds with no evidence of founder effect. It is transmitted as an X-linked dominant condition with male lethality, which usually occurs during the first and second trimester of pregnancy [7–9]. Only a small percentage of cases display familiar inheritance and the majority of mutations are sporadic (~75 %). The clinical spectrum of the disease includes craniofacial, oral and skeletal abnormalities in >80 % of cases (see [7] for details). Renal cystic disease is commonly observed as well as involvement of the CNS, which includes brain developmental anomalies and cognitive defects [10, 11]. Additional findings may include pancreatic, hepatic, and/or ovarian cysts and hearing defects [7, 12]. The gene responsible for OFD type I syndrome was identified on the short arm of the X chromosome [13]. Different mutations have been reported to date, including frameshifts, which represent the majority of mutations (>60 %), splicing, missense, nonsense and genomic rearrangements [7, 10, 11]. Additional file 1: Table S1 summarizes the mutations identified to date. The responsible gene, initially known as *CXORF5* and subsequently named *OFD1*, encodes for the centrosomal/basal body OFD1 protein [14, 15] required for left–right axis specification and for primary cilia formation [16–21]. OFD type I is a male-lethal disorder and male cases with OFD1 mutations associated to a classical OFDI phenotype have never been described. Interestingly, three affected males with “unclassified” X-linked lethal congenital malformation syndrome and a splice mutation in the *OFD1* gene have been described. The mother was mildly affected and presented only few accessory oral frenulae and irregular teeth [22]. *OFD1* mutations have also been reported in males in X-linked recessive conditions, namely (1) a mental retardation syndrome comprising macrocephaly and ciliary dysfunction [23] mapping to the same locus as Simpson–Golabi–Behemel syndrome type 2 (SGBS2); (2) Joubert syndrome (JS) patients (JBT10) [24–26] and (3) retinitis pigmentosa (RP23) [27]. These findings suggest that mutations in the OFD1 gene may result in a single syndrome spectrum characterized by wide intra- and inter-familial phenotypic variability possibly depending on the contribution of still unknown genetic modifiers.

### OFD type III syndrome (OFDIII)

OFDIII was described in 1971 [28]. Affected patients present with orofaciodigital findings similar to those described in the other OFDS, involvement of the CNS and renal disease. The typical manifestation that is only seen, among OFDs, in OFDIII cases is an oculomotor apraxia resulting in “metronome eye movements”. Recent data identified mutations in *TMEM231* (Additional file 2: Table S2) in two affected OFDIII siblings during a targeted medical sequencing of 1056 individuals with nephronophthisis-related ciliopathies [29]. The two cases presented with the typical eye movements, lingual hamartomas, postaxial polydactyly and involvement of the CNS (intellectual disabilities, cerebellar vermis hypoplasia and Dandy Walker malformation with cystic dilation of the 4th ventricle). Both cases were born with a normal renal morphology and function but developed end stage renal failure within the third decade of life. In the same report, recurrent *TMEM231* mutations were also identified in MKS patients [29]. Functional studies demonstrated that TMEM231 is involved in ciliary functions. Accordingly, mice with mutations in *Tmem231* display a clear ciliopathy phenotype including renal cystic disease, malformations of the hepatic ductal plate and skeletal abnormalities [29].

### OFD type IV syndrome (OFDIV)

This OFD subtype was originally described in a familial case in which two affected sisters displayed the typical oral-facial-digital findings in addition to severe tibial dysplasia [30, 31]. In 2012, a genome wide homozygosity mapping approach was undertaken on a case born to a consanguineous family and displaying facial dysmorphism with lobulated tongue, polydactyly of all four limbs, renal cystic disease, liver ductal plate proliferation, occipital encephalocele and other brain anomalies. X-rays examination revealed severe tibia hypoplasia and bowing of long bones. Targeted resequencing of candidate genes in homozygosity regions identified a unique nonsense mutation, c.1222C > T (p.Glu408*) in tectonic-3 (*TCTN3*). Analysis of additional cases led to the identification of three *TCTN3* truncating mutations segregating within the affected family members with the expected autosomal recessive inheritance pattern and two compound heterozygous frameshift mutations [32] (Additional file 2: Table S2). All affected cases presented with skeletal dysplasia with long bone bowing and tibia hypoplasia and only two cases displayed associated orofaciodigital findings. Interestingly, in the same study, the authors reported a *TCTN3* mutation in a JS case (c.940G > A). The JS mutation involves a nucleotide which is not affected in OFDIV patients but additional studies are required to establish a clear genotype/phenotype correlation. On the basis of these results, the authors concluded that OFDIV phenotypes can include long bone bowing, tibia hypoplasia, cystic kidney, encephalocele and other brain malformations [32].

### OFD type VI syndrome (OFDVI)

This form was initially described in 1980 in a Hungarian isolated population presenting with oro-facio-digital findings associated with central and or/cerebellar anomalies [33]. OFD VI is characterized by the presence of a “so-called” molar tooth sign (MTS) on brain MRI associated to one or more of the following: (1) hamartoma(s) of the tongue and/or additional frenula; (2) digital abnormalities (e.g. mesoaxial polydactyly of one or more hands or feet, postaxial and preaxial polydactyly) and (3) hypothalamic hamartoma. Additional oral-facial (e.g. cleft lip and palate) and/or digital signs may also be observed. The presence of the MTS allowed researchers to ascribe OFDVI to the group of Joubert syndrome (JS)-associated disorders. OFDVI differs from pure JS cases for the presence of the oral-facial-digital findings and can be defined as a rare phenotype of JS. Different studies analysed OFDVI patients and identified mutations in *TMEM216*, *OFD1*, *C5ORF42* and *TMEM107* [24, 34–37] (Additional file 2: Table S2). These findings highlight the clinical and genetic overlap among ciliopathies (see below). More recently, a comparison of *C5ORF42* mutated versus non-mutated OFDVI cases suggested a major role for this gene in limb development [38]. Interestingly, *C5ORF42* and *TMEM107* were also found mutated in pure JS cases [38]. The causality of *TMEM107* mutations in ciliopathies was confirmed by independent groups [39, 40].

### OFD type IX syndrome (OFDIX)

OFD type IX is characterized by retinal colobomata in addition to the typical oro-facio-digital findings. It is inherited as an autosomal recessive trait. Recently, a whole-exome sequencing approach identified mutations in *TBC1D32* and *SCLT1* in two patients with a severe ciliopathy phenotype [41]. Case 1 was born to healthy consanguineous parents and displayed midline defects including hypertelorism, midline clefts and severe choanal stenosis, left hand postaxial polydactyly and eye abnormalities (right microphthalmia, left anophthalmia, bilateral optic disc coloboma). Brain malformations and cardiac defects were also described. Whole-exome sequencing identified a splicing mutation in *TBC1D32* (Additional file 2: Table S2) leading to in-frame truncation of 47 amino acids. Case 2 was also born to healthy consanguineous parents and displayed severe midline cleft lip and palate, microcephaly and choanal atresia. He also presented severe coloboma and congenital heart disease. Brain malformations were also present as well as abnormal inner ear structures. In this case, exon sequencing revealed a splicing mutation in *SCLT1* resulting in complete skipping of exon 5 and the introduction of a premature stop codon (Additional file 2: Table S2). Due to the presence of the eye abnormalities, these two cases were classified as OFDIX. *TBC1D32* and *SCLT1* have both been linked to ciliogenesis. *TBC1D32* encodes a ciliary protein predicted to contain a Tre-2, Bub2 and Cdc16 (TBC) domain (TBC1D32). On the other hand, SCLT1 is an important component of the distal appendages, a centrosomal extension that establishes the connection between the mother centriole and the plasma membrane and its deficiency blocks ciliogenesis in the early phases of cilia formation [42].

### OFD type XIV syndrome (OFDXIV)

This OFD subtype was defined after the identification of mutations in the *C2CD3* gene. The first homozygous nonsense mutation (c.184C > T; p.Arg62*) was identified in a familial case in which the index case presented with classical OFD signs (lingual hamartoma, cleft and lobulated tongue, cleft palate, buccal frenulae, bilateral preaxial polydactyly of feet and postaxial polydactyly of hands) accompanied by microcephaly, micropenis and severe intellectual disabilities. Brain MRI revealed the presence of MTS, the cerebellar anomaly characteristic of JS, and other brain abnormalities (corpus callosum hypoplasia, subarachnoid cysts in the right occipital lobe and the posterior fossa, and incomplete myelination of the white matter). The presence of the MTS is intriguing and suggests a possible link with JS. His younger sister displayed a similar phenotype worsened by the presence of cardiac malformation leading to neonatal death [43]. An additional *C2CD3* compound heterozygous mutation (Additional file 2: Table S2) was identified during the screening of 34 OFD cases negative for mutations in known OFD genes in a male foetus exhibiting severe microcephaly, bilateral duplicated hallux and postaxial polydactyly, micropenis, kidney hypoplasia, corpus callosum abnormalities and inferior vermian hypoplasia with posterior cyst [43]. On the basis of these findings and of the peculiar features of microcephaly and cerebral malformations, this OFD subtype was classified as OFDXIV. Functional studies demonstrated that C2Cd3 co-localizes and physically interacts with OFD1 and is involved in centriole elongation, thus defining centriole length regulation as an emerging pathogenetic mechanism in ciliopathies [43].

### Unclassified OFDS

A number of OFD subtypes still require molecular definition and characterization of OFD patients negative for mutations in known OFD-associated genes will lead to identification also of unclassified OFD subtypes. This has already happened in the following two examples.

Whole-exome sequencing revealed compound *WDPCP* heterozygous mutations in a female child with an unclassified form of OFD [44]. This child displayed type A postaxial polydactyly of both hands and 2/3 toe syndactyly, congenital heart defects and tongue hamartomas. The two mutations (Additional file 2: Table S2) were inherited from the asymptomatic father and mother. Interestingly, mutations in the *WDPCP* transcript, which regulates planar cell polarity and ciliogenesis [45], have also been reported in a patient with Bardet–Biedl syndrome (BBS) [46], one of the first ciliopathy characterized.

Autozygosity mapping identified a minimal interval on chromosome 1 in two multiplex families of Arabian origin displaying oral (tongue lobulation, cleft palate, bifid uvula), facial (frontal bossing, midline lip defects), digital (polydactyly) signs accompanied by additional abnormalities [47]. Exome sequencing analysis identified two homozygous mutations in the *DDX59* transcript segregating with the disease in family 1 and 2 (Additional file 2: Table S2). DDX59 is highly enriched in the developing mouse palate and limb buds. Immunofluorescence analysis demonstrated a dynamic nuclear and cytoplasmic localization and normal ciliogenesis pattern in patients’ fibroblasts [47].

In both cases, future studies will clarify whether these conditions represent new OFD subtypes and identification of mutations in additional patients will be necessary to establish these two genes as bona fide ciliopathy genes.

### The link between OFDS and other ciliopathies

Mutations in ciliary genes are associated with a wide spectrum of clinical conditions that extends from viable to severe, lethal phenotypes. Oligogenic inheritance may explain this variability implying genetic interaction among different loci to cause/modulate the phenotype. This has already been shown for BBS, nephronophthisis (NPHP) and JS [48–50]. Interestingly, the unclassified OFD case with mutations in WDPCP also carried a deleterious deletion in IQCB1 which is associated to another ciliopathy, Senior–Loken syndrome, type 5 (SLSN5) characterized by early onset retinopathy and renal disease [44]. Mutations in the *OFD1* gene can be associated to a very specific phenotype as such in X-linked recessive retinitis pigmentosa (RP23) or more pleiotropic disorders such as in X-linked dominant OFD type 1, and X-linked recessive Joubert syndrome (JBTS10) and a mental retardation syndrome comprising macrocephaly and ciliary dysfunction [23]. It will be interesting to evaluate whether additional mutations in other ciliary transcript may contribute to the phenotypic outcome of *OFD1* mutations.

**Fig. 1:**
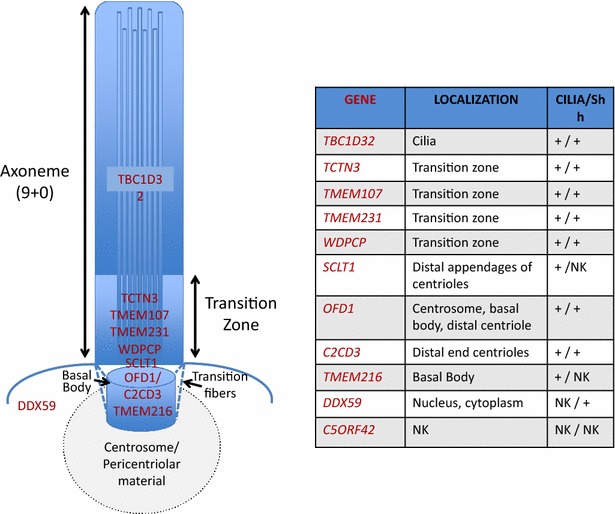
OFDS proteins map to defined cilia compartments. *Left panel,* schematic representation of primary cilia. The localization of proteins encoded by OFDS transcripts is depicted. *Right panel*, the precise cilia localization is defined. The column Cilia/Shh indicate whether a ciliary localization or perturbation of the Shh pathway has been demonstrated (+) or not (−). *NK* not known

The ciliopathy protein network can be divided in distinct but connected modules: the centrosome/basal body/pericentriolar material, the transition zone, the intraflagellar (IFT) complexes and the BBSome. OFDS genes are often mutated in other ciliopathies, especially Joubert and Meckel Gruber Syndromes. These conditions are mainly due to mutations in genes encoding proteins of the centrosome/basal body/pericentriolar material and transition zone modules (Table 3; Fig. 1) suggesting that these cilia structures have a predominant role in the pathomechanisms underlying OFDS, JS and MKS syndromes.

**Table 3:** The Involvement of OFDS transcripts in other ciliopathies

GENE^a^	CILIOPATHIES^b^
OFD1	OFDI, JBT10, RP23, SGBS2 (?)
TMEM231	OFDIII, MKS11, JBTS20
TCTN3	OFDIV, JBTS18
TMEM216	OFDVI, JBTS2, MKS2
C5ORF42	OFDVI, JBTS17
TMEM107	OFDVI, JBTS (?), MKS13
TBC1D32	OFDIX
SCLT1	OFDIX
WDCPD	BBS15, OFD unclassified, BBS12, MKS6
DDX59	OFD unclassified

^a ^Transcripts find mutated in different ^b^ ciliopathies

JBTS (?) No number has been assigned to this JBTS locus

SGBS2 (?) A mutation in the OFD1 gene was identified in affected members of a family with a X-linked mental retardation syndrome comprising macrocephaly and ciliary dysfunction. This phenotype is consistent with SGBS2 mapped to the Xp22 region

### Genes involved in OFDS: the ciliary connection

The gene responsible for OFD type I was identified in 2001 and for a while remained the only OFD gene known. In the last 3–4 years, a number of genes responsible for other OFDS have been identified mainly through next-generation sequencing (NGS) approaches. The majority of OFD genes identified localizes to cilia components (Fig. 1) and/or influence ciliogenesis with the exception of DDX59 and C5ORF52. In this section, the information available on link between genes mutated in OFDS and ciliary functions of ciliary signalling will be reviewed.

Functional studies demonstrated that OFD1 acts at the distal centriole to build distal appendages [18], thus contributing to cilia formation although in a content-specific fashion [16, 19–21]. In addition, in vivo and in vitro studies demonstrated that OFD1-depleted models show defective Shh [16, 20, 21, 51–53] and Wnt [54] signalling. In particular, impairment of Shh signalling from early stages of development may contribute to explain the skeletal malformations observed in OFDI patients.

TCTN3 and TMEM231 are components of a MKS complex localized at the transition zone of primary cilia and physically interact with each other. Functional studies demonstrated that they are both required for ciliogenesis and Shh signalling [29, 32, 55, 56].

TMEM216 localizes at the base of primary cilia and its loss results in defective ciliogenesis and centrosomal docking, with concomitant hyperactivation of RhoA and Dishevelled [35]. No information is available on the role of this transcript in Shh signalling.

TMEM107 and the planar cell polarity WDPCP proteins localize both at the transition zone, contribute to mammalian ciliogenesis [37, 45] and are required for Shh signalling [45, 57].

TBC1D32 is a ciliary protein [58] although the precise localization within cilia is not known. Functional studies demonstrate that *TBC1D32* controls ciliary morphology and is required for Shh pathway [59].

*SCLT1* and C2CD3 localize at centrioles where C2CD3 co-localizes and physically interacts with OFD1. Both proteins are necessary for ciliogenesis and C2CD3 is also required for Hedgehog signalling in mouse [42, 43, 60].

C5ORF42, also known as NKAPP1, is poorly characterized and no information is available on the role of this transcript in cilia or cilia-mediated signalling [61].

Finally, DDX59 is a member of the DEAD-box-containing RNA helicases with currently unknown function relating to cilia. Functional studies indicated that fibroblasts from affected individuals display a normal ciliogenesis pattern in the presence of reduced Shh signalling [47].

The data summarized above seem to indicate a major role for centrosomal/centriolar function in the pathomechanisms underlying OFD syndrome and components of these cilia-related cellular compartments should be considered candidate genes for the unresolved OFDS. One of the puzzling questions in OFDS as well as in other ciliopathies is how much of the phenotype is due to cilia dysfunction and how much is due to gene functions not related to cilia. Shh impairment which has been demonstrated in more than one OFDS and is linked to the ciliary function of the genes may explain the skeletal and some of the neurological findings observed. However, as we are learning by *omics* approaches, proteins may display different intracellular localization and functions [62, 63]. OFD1, for example, is localized both to centrosome/basal body and nucleus [14, 15]. Future studies will clarify the contribution of non-ciliary functions of OFD genes to the clinical spectrum of these conditions.

## Conclusions and future perspectives

A thorough clinical and molecular characterization of OFD patients will be critical to define how many subtypes do really exist for this pleiotropic condition. NGS-based approaches will define how many genes underlie OFDS and clinical studies will define how many different conditions can be clearly identified. Functional studies will clarify whether ciliopathies can be redefined not according to the genes mutated or the phenotype observed but according to the ciliary structural element functionally compromised. This knowledge may aid in designing the most appropriate approach to slow down disease progression. Finally, it is time for researchers to start studying and defining the non-ciliary functions of the transcripts mutated in OFDS (and other ciliopathies) to understand whether abnormal cilia can justify all the phenotypic abnormalities observed in OFDS.

## Additional files

10.1186/s13630-016-0034-4 Summary of mutations identified in OFDI patients.
10.1186/s13630-016-0034-4 Mutations identified in OFD genes other than OFD1.

## Abbreviations

BBS: Bardet–Biedl syndrome
NGS: next-generation sequencing
OFD: oral-facial-digital, oro-facio-digital
OFDS: oral-facial-digital/oro-facio-digital syndromes
JS: Joubert syndrome
MKS: Meckel syndrome
CNS: central nervous system
Shh: sonic hedgehog
MTS: molar tooth sign
