# Supplementary material for: Update on oral-facial-digital syndromes (OFDS)
Source: Cilia. 2016 May 2;5:12. doi: 10.1186/s13630-016-0034-4 (PMC4852435; doi:10.1186/s13630-016-0034-4)
Supplement: Supplementary file 1 — 10.1186/s13630-016-0034-4 Summary of mutations identified in OFDI patients. [file 13630_2016_34_MOESM1_ESM.docx]

Supplementary Table 2. Mutations identified in OFD genes other than OFD1

| Gene | cDNA mutation | Protein mutation | Inheritance | Diagnosis | Reference |
| --- | --- | --- | --- | --- | --- |
| TMEM231 | c.241C>T/c.373C>G | p.Leu81Phe/p.Leu125Val | mat/pat | OFD III | [1] |
| TCTN3 | c.1222C>T/ c.1222C>T | p.Gln408*/ p.Gln408* | pat/mat | OFD IV | [2] |
| TCTN3 | c.650_653del/ c.650_653del | p.Tyr217Serfs*6/ p.Tyr217Serfs*6 | pat/mat | OFD IV | [2] |
| TCTN3 | c.650_653del/ c.650_653del | p.Tyr217Serfs*6/ p.Tyr217Serfs*6 | pat/mat | OFD IV | [2] |
| TCTN3 | c.1357C>T/c.1357C>T | p.Gln443*/p.Gln443* | pat/mat | OFD IV | [2] |
| TCTN3 | c.566_567del/ c.1348_1349del | p.Glu189Valfs*52/ p.Leu450Serfs*14 | pat/mat | OFD IV | [2] |
| TCTN3 | c.940G>A/ c.940G>A | p.Gly314Arg/ p.Gly314Arg | pat/mat | OFD IV | [2] |
| TMEM216 | c.218G>T/ c.218G>T | p.Arg73Leu/ p.Arg73Leu | mat/pat | OFD VI | [3] |
| TMEM216 | c.218G>T/ c.218G>T | p.Arg73Leu/ p.Arg73Leu | mat/pat | OFD VI | [3] |
| C5orf42 | c.3557delA/ c.3577C>T | p.Lys1186Argfs*21/ p.Arg1193Cys | NA | OFD VI | [4] |
| C5orf42 | c.3550C>T/ c.9121C>T | p.Arg1184Cys/ p.Gln3041* | pat/mat | OFD VI | [4] |
| C5orf42 | c.3150-1G>T/ c.3150-1G>T | NA/NA | pat/mat | OFD VI | [4] |
| C5orf42 | c.493delA/ c.3290-2A>G | p.Ile165Thrfs*17/NA | pat/mat | OFD VI | [4] |
| C5orf42 | c.2377C>T/ c.8509G>T | p.Gln793*/ p.Val2837Leu | pat/mat | OFD VI | [4] |
| C5orf42 | c.493delA/ c.3380C>T | p.Ile165Thrfs*17/ p.Ser1127Leu | pat/mat | OFD VI | [4] |
| C5orf42 | c.3380C>T/ c.3859G>C | p.Ser1127Leu/ p.Asp1287His | pat/mat | OFD VI | [4] |
| C5orf42 | c.3859G>C/ c.7476delT | p.Asp1287His/ p.Arg2493Aspfs*6 | NA | OFD VI | [4] |
| C5orf42 | c.6898delC/ c.7402C>T | p.Gln2300Argfs*8/ p.Gln2468* | NA | OFD VI | [4] |
| C5orf42 | c.677A>G/ c.677A>G | p.Gln226Arg/ p.Gln226Arg | mat/pat | OFD VI | [5] |
| TMEM107 | g.8079298T > C | p.Glu45Gly | mat/pat | OFD VI | [6] |
| SCLT1 | c.290+2T>C/ c.290+2T>C | p.Lys79Valfs*4/ p.Lys79Valfs*4 | mat/pat | OFD IX | [7] |
| TBC1D32 | c.1372+1G>T/ c.1372+1G>T | p.Arg411_Gly458del/ p.Arg411_Gly458del | mat/pat | OFD IX | [7] |
| C2CD3 | c.184C>T/ c.184C>T | p.Arg62*/ p.Arg62* | mat/pat | OFD XIV | [8] |
| C2CD3 | c.3085T>G/ c.3911-2A>T | p.Cys1029Gly/p.Ala1304Valfs*3 | NA | OFD XIV | [8] |
| DDX59 | c.1600G>A/ c.1600G>A | p.Gly534Arg/ p.Gly534Arg | mat/pat | Unclassified OFD | [9] |
| DDX59 | c.1100T>G/ c.1100T>G | p.Val367Gly/ p.Val367Gly | mat/pat | Unclassified OFD | [9] |
| WDPCP | c.552_553del/ c.160G>A | p.Cys185Phefs*12/ p.Asp54Asn | pat/mat | Unclassified OFD | [10] |

REFERENCES

1. Roberson EC, Dowdle WE, Ozanturk A, Garcia-Gonzalo FR, Li C, Halbritter J et al. TMEM231, mutated in orofaciodigital and Meckel syndromes, organizes the ciliary transition zone. J Cell Biol. 2015;209(1):129-42. doi:10.1083/jcb.201411087.

2. Thomas S, Legendre M, Saunier S, Bessieres B, Alby C, Bonniere M et al. TCTN3 Mutations Cause Mohr-Majewski Syndrome. Am J Hum Genet. 2012;91(2):372-8. doi:10.1016/j.ajhg.2012.06.017.

3. Valente EM, Logan CV, Mougou-Zerelli S, Lee JH, Silhavy JL, Brancati F et al. Mutations in TMEM216 perturb ciliogenesis and cause Joubert, Meckel and related syndromes. Nat Genet. 2010;42(7):619-25. doi:10.1038/ng.594.

4. Lopez E, Thauvin-Robinet C, Reversade B, Khartoufi NE, Devisme L, Holder M et al. C5orf42 is the major gene responsible for OFD syndrome type VI. Hum Genet. 2014;133(3):367-77. doi:10.1007/s00439-013-1385-1.

5. Bayram Y, Aydin H, Gambin T, Akdemir ZC, Atik MM, Karaca E et al. Exome sequencing identifies a homozygous C5orf42 variant in a Turkish kindred with oral-facial-digital syndrome type VI. Am J Med Genet A. 2015;167(9):2132-7. doi:10.1002/ajmg.a.37092.

6. Lambacher NJ, Bruel AL, van Dam TJ, Szymanska K, Slaats GG, Kuhns S et al. TMEM107 recruits ciliopathy proteins to subdomains of the ciliary transition zone and causes Joubert syndrome. Nat Cell Biol. 2015. doi:10.1038/ncb3273.

7. Adly N, Alhashem A, Ammari A, Alkuraya FS. Ciliary genes TBC1D32/C6orf170 and SCLT1 are mutated in patients with OFD type IX. Hum Mutat. 2014;35(1):36-40. doi:10.1002/humu.22477.

8. Thauvin-Robinet C, Lee JS, Lopez E, Herranz-Perez V, Shida T, Franco B et al. The oral-facial-digital syndrome gene C2CD3 encodes a positive regulator of centriole elongation. Nat Genet. 2014;46(8):905-11. doi:10.1038/ng.3031.

9. Shamseldin HE, Rajab A, Alhashem A, Shaheen R, Al-Shidi T, Alamro R et al. Mutations in DDX59 implicate RNA helicase in the pathogenesis of orofaciodigital syndrome. Am J Hum Genet. 2013;93(3):555-60. doi:10.1016/j.ajhg.2013.07.012.

10. Saari J, Lovell MA, Yu HC, Bellus GA. Compound heterozygosity for a frame shift mutation and a likely pathogenic sequence variant in the planar cell polarity-ciliogenesis gene WDPCP in a girl with polysyndactyly, coarctation of the aorta, and tongue hamartomas. Am J Med Genet A. 2015;167A(2):421-7. doi:10.1002/ajmg.a.36852.
