# Supplementary material for: Update on oral-facial-digital syndromes (OFDS)
Source: Cilia. 2016 May 2;5:12. doi: 10.1186/s13630-016-0034-4 (PMC4852435; doi:10.1186/s13630-016-0034-4)
Supplement: Supplementary file 2 — 10.1186/s13630-016-0034-4 Mutations identified in OFD genes other than OFD1. [file 13630_2016_34_MOESM2_ESM.pdf]

Supplementary Table 1. Summary of mutations identified in OFDI patients

Exon/intron	Nucleotide change	Type of mutation	Predicted protein	Total number of index cases
Intron 1	c.13-10T>A	Splice site		1
Exon 2	c.43_44delAG	Frameshift	p.Q16RfsX17	1
	c.65dupA	Frameshift	p.L23AfsX28	1
	c.111G>A	Splice site		1
	c.111G>C	Splice site		1
	c.63insT	Splice site	p.lys21Aspfs*8	1
	c.52G>T	Nonsense	p.Glu18*	1
Intron 2	c.111+2T>C	Splice site		2
	c.111+3A>G	Splice site		1
Exon 3	c.121C>T	Nonsense	p.R41X	2
	c.162_166delTGGAG	Frameshift	p.S54RfsX73	1
	c.221C>T	Missense	p.S74F	2
	c.224A>C	Missense	p.N75T	1
	c.235G>A	Missense	p.A79T	1
	c.241C>G	Missense	p.H81D	1
	c.243C>G	Missense	p.H81Q	1
	c.247C>T	Nonsense	p.Q83X	1
	c.260A>G	Missense	p.Y87C	1
	c.274T>C	Missense	p.S92P	1
	c.290A>G	Missense	p.E97G	1
	c.294_312del TGGTTTGGCAAAAGAAAG	Frameshift	p.S98RfsX138	1
	c.312delG	Frameshift	p.V105YfsX144	1
	c.313dupG	Frameshift	p.V105GfsX116	1
	c.148insG	Frameshift	p.His50A1afs*26	1
	c.275_276delCT	Frameshift	p.ser92Cyssf*24	1
Intron 3	c.312+2_312+8delAAAGTC	Splice site		1
Exon 4	c.337C>T	Nonsense	p.Q113X	1
	c.372C>G	Nonsense	p.Y124X	1
	c.412G>A	Missense	p.G138S	1
Intron 4	c.382-3C>G	Splice site		1
	c.382-2A>G	Splice site		1
Exon 5	c.400_403delGAAA	Frameshift	p.E134IfsX1	3
	c.411delA	Frameshift	p.G138VfsX144	1
Intron 5	c.412+2delT	Splice site		1
	c.413-10T>G	Splice site		1
Exon 6	c.431dupT	Frameshift	p.L144FfsX154	1
	c.431T>A	Nonsense	p.L144X	1
	c.454C>T	Nonsense	p.Q152X	1
	c.422T>G	Missense	p.Met141Arg	1
	c.508_509delGA	Frameshift	p.Asp170Phefs*4	1
Intron 6	c.518-1G>A	Splice site		1
Exon 7	c.594_598delAAAGC	Frameshift	p.L200X	1
	c.602delA	Frameshift	p.N201MfsX207	1
	c.615_620delAGAAAT	In-frame deletion	p.E606_I607del	1
	c.616_617delGA	Frameshift	p.E206NfsX222	1
	c.628C>T	Nonsense	p.Q210X	1
	c.653delA	Frameshift	p.K218SfsX219	1
	c.541dupG	Frameshift	p.Asp181Glyfs*22	1
Intron 7	c.654+2_654+4delTA	Splice site		1
	c.2388+1G > C	Splice site		1

Exon/intron	Nucleotide change	Type of mutation	Predicted protein	Total number of index cases
Exon 8	c.675delC	Frameshift	p.E226RfsX227	1
	c.702insA	Frameshift	p.Y238VfsX239	1
	c.707_719delAAAAGTATGAAAA	Frameshift	p.K236RfsX238	1
	c.709_710delAA	Frameshift	p.K237VfsX238	1
	c.710delA	Frameshift	p.K237SfsX242	2
	c.710dupA	Frameshift	p.Y238VfsX239	7
	c.712delT	Frameshift	p.Y238MfsX242	1
	c.790dupG	Frameshift	p.E264GfsX269	1
	c.823C>T	Nonsense	p.Q275X	1
	18-bp deletion	In-frame deletion	p.230-235del IKMEAK	1
Exon 9	c.837_838delAA	Frameshift	p.K280RfsX307	2
	c.837_841delAAAAG	Frameshift	p.K280NfsX306	1
	c.839_840delAA	Frameshift	p.K280RfsX307	1
	c.843_844delAA	Frameshift	p.E281DfsX307	1
	c.858delG	Frameshift	p.R286SfsX290	1
	c.871A>T	Nonsense	p.K291X	1
	c.877_878delAT	Frameshift	p.M293GfsX307	2
	c.895insGA	Frameshift	p.A310KfsX304	1
	c.919delG	Frameshift	p.V307LfsX312	1
Intron 10	c.1051-2>G	Splice site		1
	c.1056-2A>T	Splice site		1
Exon10a	c.1056C>G	Missense	p.N352K	1
Exon11	c.1071_1078 del GAAGGATG/ins TTTTTCCT	Missense	p.KDD 357_359del/FSY 357_359ins	1
	c.1099C>T	Nonsense	p.R367X	1
	c.1100G.>A	Missense	p.R367Q	1
Intron 11	c.1130-20_1130-17delAATT	Splice site		1
Exon12	c.1178dupA	Frameshift	p.E394GfsX407	1
	c.1185del A	Frameshift	p.E395DfsX400	1
	c.1193_1196delAATC	Frameshift	p.Q398LfsX400	4
	c.1220_1221+1delAGG	Frameshift	p.E407AfsX408	1
Intron 12	1221+1delG	Splice site		1
Exon13	c.1268_1272delAAAAC	Frameshift	p.Q423PfsX428	2
	c.1303A>C	Missense	p.S434R	1
	c.1318delC	Frameshift	p.L440X	1
	c.1319delT	Frameshift	p.L440QfsX469	1
	c.1322_1326delAAGAA	Frameshift	p.K441RfsX450	1
	c.1323_1326delAGAA	Frameshift	p.E442RfsX468	1
	c.1334_1335delTG	Frameshift	p.L445RfsX451	1
	c.1358T>A	Nonsense	p.L453X	1
	c.1360_1363delCTTA	Frameshift	p.L454NfsX468	1
	c.1409delA	Frameshift	p.N470TfsX472	1
Exon 14	c.1420C>T	Nonsense	p.Q474X	1
	c.1445_1446delTT	Frameshift	p.F482SfsX495	1
	c.1452_1458delAGAACTA	Frameshift	p.K484NfsX491	1
Exon 15	c.1587delA	Frameshift	p.A530LfsX532	1
	c.1612C>T	Nonsense	p.Gln538*	1
Exon 16	c.1757delG	Frameshift	p.S586MfsX590	1
	c.1821delG	Frameshift	p.I608SfsX628	1
	c.1887_1888insAT	Frameshift	p.N630IfsX666	1

Exon/intron	Nucleotide change	Type of mutation	Predicted protein	Total number of index cases
	c.1979_1980delCT<br>c.2044dupA<br>c.2056delT<br>c.2122-2125dupAAGA<br>c.2176delC<br>c.1859_1860delC<br>c.1990dupC	Frameshift<br>Frameshift<br>Frameshift<br>Frameshift<br>Frameshift<br>Frameshift<br>Frameshift	p.S660CfsX<br>p.I682NfsX700<br>p.S686PfsX717<br>p.N711KfsX713<br>p.R726AfsX516<br>p.Ser620Cysfs*8<br>p.Leu665Thrfs*35	3<br>1<br>1<br>1<br>1<br>1<br>1
Intron 16	c.2261-1G>T	Splice site		1
Exon 17	c.2349delC	Frameshift	p.I784SfsX816	1
Deletion	c(?_-311)_828+?del	Deletion exons 1-8		1
Deletion	c(?_-311)_1542+?del	Deletion exons 1-14		1
Deletion	c381-?_412+?del	Deletion exon 5		1
Deletion	c936-?_1129+?del	Deletion exons 10-11		1
Deletion	c1222-?_3038+?del	Deletion exons 13-23		1
Deletion	c2261-?_2387+?del	Deletion exon 17		1
Deletion	c518-?_935+?del	Double Deletion (exons 7-9 and 14bp in intron 9		1
Deletion spanning the whole OFD1 gene		Large deletion		1
Deletion spanning the whole OFD1 gene		Large deletion		1

*As reference, the A of the ATG translation initiation start site of the coding sequence for *OFD1* (Entrez nucleotide accession number NM_003611) is referred to as nucleotid
